# Round the Hospitals

**Published:** 1919-03-29

**Authors:** 


### ROUND THE HOSPITALS.

At the Investiture held in the Ball-room of Buckingham Palace on March 20, the King personally conferred the decoration of the Royal Red Cross, First Class, on Sister Jessie Spittal, T.F.N.S., and on Sister Marion Ruddick and Sister Catherine Scoble, both of the Canadian A.N.S. His Majesty conferred the Second Class of the Order on Sister Margaret McLean, Sister Gertrude Smith, and Sister Jane Walker, of the T.F.N.S.; on Sister Elizabeth Cameron, Sister Annie Mitchell,

**ROUND THE HOSPITALS**—(*continued*).

and Sister Katharine Reid, of the Canadian A.N.S. The members of the Military Nursing Services who had received these decorations had the honour of being received subsequently at Marlborough House by Queen Alexandra.

At the Investiture held on March 22, the King personally conferred the Royal Red Cross, First Class, on Matron Jean Urquhart and Matron Evelyn Wilson, both of the Canadian A.N.S. His Majesty conferred the Second Class of the Order on Staff Nurse Annie Boyd, Q.A.I.M.N.S.R.; Sister Amy Bevan and Sister Florence Henderson, T.F.N.S.; on Matron Sadie McIsaac, Sister Margaret Macdonald, and Sister Alexandra Nelson, of the Canadian A.N.S. Queen Alexandra received the ladies at Marlborough House after the Investiture.

The news that the Nurses' Registration Bill of the Central Committee has drawn a good place in the ballot and will come before the House of Commons on March 28 affects very deeply the interests of all nurses. We may say at once that no measure which is not supported by the great body of expert and authoritative nursing opinion has any chance of becoming law. For this reason there is an opening for further negotiation to obtain an agreed Bill and bring about something like unanimity on the subject. Whether this be possible or not it is too early to say, but at the time of going to press the efforts were continuing in a hopeful spirit. The Member of Parliament in charge of the College of Nursing Bill is Lt-Colonel Nathan Raw, C.M.G., R.A.M.C., who represents Liverpool (Wavertree). We publish on page 576 a clear statement issued by the College of Nursing which will enable nurses to see the distinction between the two Bills.

It is very important that trained nurses should co-operate to the utmost of their ability with the Salaries Committee appointed by the College of Nursing to examine into and report on "conditions of employment and salaries." All who approach its Secretary, whether personally or by letter, to offer first-hand information, may rely upon their communications being regarded as confidential. On and after March 31 a member of the Salaries Committee will be in attendance within office hours at the College of Nursing, 7 Henrietta Street, Cavendish Square, W. 1, and will welcome any who can throw light on the subjects under consideration. Letters should be addressed to the Secretary of the Salaries Committee as above.

Alterations have been made in the sisters' salaries at St. Bartholomew's which make these posts some of the most desirable in the nursing world. As is well known, the sisters in this institution hold unique status and tend to become permanent controllers of their wards, subject, of course, to the authority of the matron. They are provided with dinner only by the hospital, so that some part of their salary is to be regarded as messing allowance. At the same time, they receive an additional 6s. a week in consideration of the war price of provisions. Their rate of pay has now been raised from £65 (rising by quinquennial increment of £6 10s. to £84 10s.) to £75, increasing £5 every three years to £90. The assistant matron is now to receive £90, and rise by increment of £5 every three years to £120; the superintendent of the Nurses' Home, £75—£90; house sister, £50—£60; office sister, £55—£60; night superintendents, £55; theatre sister, £75—£90; out-patient sister, £80—£90; superintendent of Special Probationers' Home, £65 to £80. In all cases the rise is at the rate of £5 every three years. Certificated nurses, remaining after completion of their contract, receive £40 in their fifth and subsequent years. A new and grateful order taking effect from March 3 entitles all staff nurses, staff probationers, and probationers to a sleeping-out pass when they have an evening off duty preceding a long day. Concessions have also been made to probationers enabling them to get a late pass till 10 P.M. on long days and half-days, and to late passes once a month or once a fortnight, according to grade.

During the past year the salaries at the Beckett Hospital, Barnsley, have been increased as follows: the matron to £125; the assistant-matron to £70; massage sisters to £70; night-sisters to £55; day sisters to £50; staff nurses to £35. The sisters are provided with inside uniform. Probationers' salaries have been increased to £12, £16, and £20, with outdoor and indoor uniforms free. The staff has two hours off duty each day, one half day holiday each week, and one full day each month, also one month's holiday each year. The probationers have similar times off, and three weeks' holiday yearly. The increase of the present salaries over those given in 1914 amounts to a total sum of £425 3s.

Salaries at the City Hospital, Leeds, have been raised by the Town Council as follows: Home sister, £70; night and ward sisters, £50, rising by £2 to £60; staff nurses, £40 to £48; probationers, £20 to £25.

At the Leeds Poor-Law Infirmary great reductions have been made in the hours of duty for the nursing staff. Day nurses, whose working hours averaged nine and a-half, will now have an average eight-hour day, working 55¼ hours weekly. The sisters, whose week now comprises fifty-five hours on duty, are to have one whole day off once a week, and by this their hours are reduced to fifty in the week. The principal alteration in the night nurses' hours is that they get one night off per week instead of two a month. The matron reported that "probably the night nurses sit down two half-hours for meals during the night." She would like to give them a hot meal in the night and an hour's rest in the mess-room when more accommodation can be provided. Twelve more nurses will be required

**ROUND THE HOSPITALS—(*continued*).**

by the eight-hour day scheme. We note also that an eight-hour day for the nurses has been introduced at the North Bierley Infirmary.

The members of Queen Alexandra's Imperial Military Nursing Service Reserve who wrote to the *Times* complaining of being "dismissed summarily," have been answered. They did not take the trouble to verify their facts, and are convicted of more than one mis-statement. Their second letter does not better their case. For instance, these nurses say "the extra £20 added to our salaries in September 1916 had to be refunded," whereas the authorities aver that all who signed a contract in November 1916 to serve for as long as required received £20 a year extra pay, and that no nurse demobilised by the War Office has been required to refund this. Again, the nurses say that a month's notice is required from them, but this is stated not to be the case. Unless nurses contracted to remain on at a higher salary they have been at liberty to resign and quit the Reserve as soon as their papers were passed. Again, these nurses indignantly ask whether they ought not to receive "one month's wages and allowances" on quitting the service, the truth being that having the status of officers they receive a gratuity of £7 10s. in the case of a staff nurse, £10 in the case of a sister, and £15 in the case of a matron for each year's service in lieu of such wage and allowance. In the case of nurses who claim to have worked four years these gratuities would amount to £30, £40, and £60 respectively. If ill or unfit for work they can go before a sick board and get sick leave with pay before demobilisation. Most Q.A.I.M. nurses are grumbling at the delay in demobilising them, and it is to meet the extraordinary demand for their services that the fourteen days' leave, which may happen to fall due prior to demobilisation, has been abrogated. We are glad to hear that the Financial Secretary has it under consideration to increase the scale of the gratuity.

A remarkable and valuable member of the nursing service of this country passed away on March 20 at the Preston Royal Infirmary, in the person of Miss Jessie Gibson, who has fallen a victim to the influenza epidemic. She was born in Elgin, and it was there that she was buried, a memorial service being held at the hospital. Sister Gibson was one of the first nurses trained under the three years' system at the Preston Royal Infirmary. She was promoted sister of the small wards in 1896, and held that post till her death. She was a woman of strong personality, with a very forcible influence for good. She was imbued with a beautiful loyalty to the hospital and her superiors in office. As ward-sister she was a strict disciplinarian, redoubted by those under her jurisdiction, but a firm friend. All who came under her sway learned to love her, and none ever forgot her. Former nurses and former house-surgeons found time to write to Sister Gibson from distant parts of the world, and she had the great gift of making herself live in her answering letters. Though she spent all her life in the one hospital she loved, she was not narrow-minded, but alive to the world of books, music, and politics. Her thrifty influence set many a probationer on the path of saving for old age. She will be sorely missed by the matron, Miss Marks, and by all her colleagues. It may be added that her gifts as a ward sister were recognised and publicly mentioned by Sir Henry Burdett, K.C.B., in the course of an inspection of the Royal Infirmary, Preston, in 1914. He said of her: "She has her heart and soul in the work. She is as happy as a queen; she has all the people under her happy too."

Can any one credit the statement made by Mr. Lyle in the House of Commons that the number of trained nurses left in the kingdom amounts to little more than the 20,000 engaged in naval and military hospitals? Our reasons for scepticism on this point are grounded on the fact that hardly any hospital or nurses' institute in the country has surrendered as many as half its nurses for military duty. In most cases, and we have had wide opportunities for knowing the facts, only a fourth of the staff have been able to go, often only a small fraction of the whole, though the hardship to the hospitals lay in the fact that it was those least able to be spared who obeyed the call. For every nurse who went there must certainly have been three left at home. Many returned after a year or two of war work and set free others. Again, the war has lasted nearly five years, and during that time at least 3,000 newly-trained nurses have been turned out annually from hospitals and Poor-Law infirmaries. It can be seen by studying reports that only a fraction of the newly-trained took up military nursing; the majority have taken posts in institutions or as district and private nurses, much as usual. Reckoning for 10 per cent. wastage through marriage, illness, etc., there would still be over 13,000 probationers trained during the war, of whom quite 8,000 must be on home duty. Taking those facts together we believe the number of trained nurses altogether cannot be far short of 100,000, though not all may be following their calling at any one time.

A gala day was held on March 20 at Queen Alexandra's Hospital for Officers, Highgate, to celebrate the fourth anniversary of its opening. The Right Hon. Sir Alfred Mond, M.P., chairman of the hospital, presided, and presented the matron, Miss Sinzininex, and each member of the staff, with a wrist watch in appreciation of their admirable work. The hospital now contains fifty-seven beds. It is situated on the southern slope of Highgate Hill, and the conditions of pure air and complete quiet have produced a markedly beneficial effect in speeding up recovery. The hon. surgeon in charge, Mr. H. J. Paterson, reports that the highest possible standard of nursing has been maintained throughout the four years of work.

**ROUND THE HOSPITALS—(*continued*).**

Some rumour of the deplorable dispute which took place last year between ten sisters at the Scottish Hospital at Rouen and the matron, Miss Riddell, has no doubt penetrated to many of our readers. It ended in the decision of the Scottish Branch of the British Red Cross Society to recall the ten sisters, and they have now, under the guidance of their advocate, Mr. G. W. Wilton, put forward a printed document containing their appeal and case, with copies of the letters which have passed on both sides. It appears that on January 13, 1918, nine sisters, believing the matron to be on the point of reporting Miss Johnstone, the theatre sister, to headquarters with a view to her dismissal, wrote a letter of protest against the matron and addressed it to the Committee of the B.R.C.S., Scottish Branch. It is not a good letter. It displays intense animus against the matron and states that "for a very considerable time the staff have had it in their minds to lodge a formal complaint with the Committee on the ground of Miss Riddell's behaviour to them." Such a statement goes far in our view to explain Miss Riddell's counter complaint of want of loyalty in her staff. No serious allegations against character or professional competence were made by either the matron or the sisters against each other. But the sisters protested that Miss Riddell had "abused her position as matron." Is it possible that they did not apprehend the gravity of making a charge of this description against their superior officer?

The sisters have not been fortunate in their advocate. Truth to tell, there is a great deal too much of Mr. Wilton in the case from first to last, and his epistolary style does not make a good impression. It is nothing short of a tragedy that through faults of temper and manner the admirable work which was being accomplished at the Rouen Scottish Hospital should have been endangered, and women doing good service in their respective departments led into variance and mutual recrimination. And this in the very worst part of the war, when every available ounce of energy was required. We feel sure that some of the sisters, if not all, now bitterly regret that they did not hold on in silence for the few remaining months of their service.

The account we are able to publish this week of the projected Nurses' Home at St. Bartholomew's will reassure all admirers of this historic establishment who may have been found doubting whether even yet the governors were prepared to rise to the height of the situation in respect of the housing of their nurses. That the proposed Nurses' Home will be worthy of the building which for 800 years has enjoyed the benefit of women's ministrations in its wards is no light thing to say, but so far as money, zeal, and knowledge can go in realising a great deal this purpose will be fulfilled. The triumphs of the surgeon are closely dependent on the faithfulness, skill, and well-being of the nurse. Let the public but fully understand this and the necessary funds for St. Bartholomew's Nurses' Home will pour in without delay.

Sister Charlton, R.R.C., has for four years been the only trained sister at the Red Cross Hut at Chelmsford, and during this time 1,900 wounded have passed through her hands. Her staff was made up of members of V.A.D. Essex 40, under Miss Kemble, M.B.E., the efficient Commandant. Sister Charlton received numerous gifts from all who had been associated with her in the work, and has now sailed for Serbia with a unit in connection with Lady Grogan's Serbian Relief Fund.

People are less inclined to smile than they once were when we used to say that a day would come when better accommodation would be required for ward maids than was then considered sufficient, and even generous, for nurses. Since then up-to-date hospitals possess nurses' homes, which it really would not be unfair to compare to the "first-rate hotels" so preposterously promised to the insured consumptive. The standard of the new nurses' homes has, as we prophesied, begun to raise the standard of the ward maids' quarters. The latest instance is that of the authorities of the Liverpool Royal Infirmary, who, in proposing to spend £70,000 on a new nurses' home, expressly include "more roomy quarters" for ward maids. We remember one Midland hospital where the servants, just before the war, were sleeping in what was virtually a series of cellars. Perhaps they are still; but when all such abominations have been removed we hope that the ward maid will have become, in her turn, improved also. For even the world of labour, irrespective of sex, has something to learn as well as something to complain of.

Great searchings of heart have arisen at a Wellington, New Zealand, hospital board meeting by reason of a proposed by-law empowering the matron to suspend a nurse for "incapacity, disobedience, neglect of duty, etc." The members of the board, by one vote, decided against entrusting the matron with this very necessary authority over her subordinates, although in the model by-laws prepared for the assistance of hospital boards by the Public Health Department it is laid down that the matron shall have power to suspend any nurse, reporting the same to the medical officer, who shall in turn report the matter to the board. Business men probably think that a delay of a few days in suspending a nurse is of slight importance. Bad work done in such an interval, for instance, by a neglectful clerk could be put right afterwards. But suppose a defiant or neurotic nurse had to be left in charge of patients pending the decision of the board to relieve her of her duties, is it not clear that the lives of the sick and the reputation of the hospital would be involved? The matter is one of such great importance that we trust the promoters of the reform will persevere till they gain the day.

We are asked to note that the staff at the Ancoats Hospital, Manchester, now consists of thirty-two nurses and nine sisters. To allow for the one day in seven leave three additional nurses have been engaged.

